# Equity-Related Determinants of Frequent Emergency Department Visits for Substance Use Disorders: A Population-Based Study in Ontario, Canada

**DOI:** 10.1177/00207640261419135

**Published:** 2026-02-28

**Authors:** Soyeon Kim, Kenneth P. Fung, Natalie Rajack, Florence Tang, Aditi Patrikar, Kinwah Fung, Erik L. Friesen, Paul Kurdyak, Bernard Le Foll

**Affiliations:** 1 Department of Psychiatry and Behavioural Neurosciences, Faculty of Health Sciences, McMaster University, Hamilton, ON, Canada; 2 Waypoint Research Institute, Waypoint Centre for Mental Health Care, Penetanguishene, Canada; 3 Department of Psychiatry, Temerty Faculty of Medicine, University of Toronto, Canada; 4 Centre for Mental Health, Toronto Western Hospital, University Health Network, Toronto, ON, Canada; 5 Faculty of Social Sciences and Humanities, Ontario Tech University, Oshawa, Canada; 6 Mental Health and Addictions Research Program, Institute for Clinical Evaluative Sciences (ICES), Toronto, ON, Canada; 7 Translational Addiction Research Laboratory, Campbell Family Mental Health Research Institute, Center for Addiction and Mental Health, Toronto, Canada

**Keywords:** substance use, marginalization, emergency department visit

## Abstract

**Background:**

Substance use disorders (SUDs) represent a growing public health concern in Canada, contributing to emergency department (ED) overcrowding and high system costs. Despite rising rates of SUD-related ED visits, the role of equity-related determinants, such as socioeconomic status, households and dwellings marginalization, and access to coordinated care, remains insufficiently understood.

**Aim:**

This study examined how equity-related and sociodemographic factors, along with clinical and service utilization characteristics, are associated with frequent ED visits for SUD in Ontario, Canada.

**Methods:**

Using provincial health administrative data from ICES, we conducted a retrospective cohort analysis of individuals aged 12 and older with an SUD-related ED visit between April 2022 and March 2023. Frequent ED visits were defined as three or more substance use–related emergency department visits in the 12 months preceding an individual’s index visit, excluding the index visit itself, which represented approximately the 90th percentile of visit frequency in the cohort. Logistic regression identified factors associated with frequent ED utilization (⩾3 annually).

**Results:**

Frequent SUD-related ED visitors were disproportionately young adults (25–44 years) and male. Individuals in the most housing-unstable areas had significantly higher odds of frequent ED visits. Racialized and newcomer indices were not statistically associated with frequent visits. Comorbid mental illness, chronic diseases, and alcohol use were strong clinical correlates. High service utilization patterns, including prior mental health and acute care hospitalizations, were also associated with frequent ED use.

**Conclusion:**

High-frequency ED users with SUD reflect a convergence of clinical complexity, socioeconomic vulnerability, and systemic gaps. While households and dwellings marginalization emerged as a key factor, the absence of associations with racialized or newcomer indices should not be misread as a lack of need, as these indices were measured at the area level. These findings highlight the urgent need for equity-informed, integrated care to reduce preventable ED use.

## Introduction

Substance use disorders (SUD) cause an increasingly significant burden of disease globally (Degenhardt et al., 2018). In Canada, SUD leads to 200 deaths every day, and in 2020, it was estimated to have cost $49 billion (i.e., due to expenses related to healthcare, lost productivity, criminal justice, and other direct costs; Canadian Substance Use Costs and Harms Scientific Working Group, 2023). However, two-thirds of people in Canada with SUD do not receive adequate care, leading to significant unmet healthcare needs (Urbanoski et al., 2017). Consequently, nearly 1 in 11 emergency department (ED) patients were identified as having an SUD, which plays a significant role in ED overcrowding (Moe et al., 2022). ED overcrowding remains among the most challenging issues facing the Canadian healthcare system. It contributes to decreased quality of care and increased likelihood of medical error, and it is an expensive form of medical care (Canadian Institute for Health Information, 2025; Owen et al., 2010; Rader & Ritchie, 2023).

Numerous studies have examined the characteristics of individuals who frequently visit the ED to identify those requiring more support and inform policy strategies accordingly. However, research on frequent ED patients with SUD has been constrained, partly due to limited data availability. Some studies have reported on general demographic characteristics (e.g., age, sex; Fleury et al., 2019; Kim, Weekes et al., 2023; Lavergne et al., 2022; Moe et al., 2022) and healthcare-related variables (e.g., hospital characteristics, the type of substance used; Fleury et al., 2019; Kim, Weekes et al., 2023; Lavergne et al., 2022; Moe et al., 2022). Recently, Fleury et al. (2024) identified distinct profiles of repeat and very high ED users in a Canadian cohort, revealing that these groups present with more severe conditions, require more outpatient care, and face higher risks of hospitalization and death. Nonetheless, in the context of SUD, a condition often shaped by systemic and structural inequalities, understanding the impact of equity-related factors on treatment outcomes and care pathways is particularly crucial (Bell et al., 2024; Lin et al., 2024). While some research has incorporated measures of material and social deprivation as proxies for marginalization (Armoon et al., 2021; Pampalon et al., 2009; Penzenstadler et al., 2020) among SUD patients, other critical dimensions of equity, such as racialized identity, newcomer status, or education level, remain understudied, despite their potential role in shaping patterns of ED use. This is especially concerning given that service access disparities tend to be most pronounced among older adults, ethnocultural minorities, immigrants, and individuals with lower socioeconomic status (Krieg et al., 2016; Urbanoski et al., 2017; Williamson, 2024). Further investigation into these equity-related factors is necessary to better understand their role in shaping SUD-related ED use. Similarly, healthcare costs associated with previous utilization patterns have been largely overlooked in existing research (Armoon et al., 2021; Crispo et al., 2023; Friesen et al., 2023). Nevertheless, cost-related data are critical for identifying high-need, high-cost subgroups and assessing where health system resources are disproportionately consumed. Frequent ED visitors for SUD often account for a disproportionate share of healthcare expenditures due to repeated acute care visits and associated diagnostic or treatment services (Beckerleg & Hudgins, 2022). This information enables the development of more precise, population-tailored interventions that not only reduce unnecessary ED use but also address upstream social and structural determinants of health. In turn, such targeted approaches can improve care continuity, reduce health disparities, and enhance the economic sustainability of healthcare systems.

This study sought to address the aforementioned critical research gaps by identifying key sociodemographic, equity-related, clinical, and healthcare utilization factors associated with frequent ED visits for SUD. The study provides a comprehensive perspective on SUD ED visits within the North American context by analyzing multiple integrated health administrative datasets from across Ontario.

## Methods

### Study Design and Dataset

This cross-sectional study used retrospective health administrative data from Ontario stored at ICES, an independent, not-for-profit research institute authorized under Ontario’s Personal Health Information Protection Act (PHIPA) to analyze de-identified health data without individual consent (Schull et al., 2020). Under the PHIPA, this study is authorized to use the collected data without Research Ethics Board approval. Data for this study are held securely at ICES and are not publicly available, but may be accessed by eligible researchers through ICES under approved protocols. Datasets used include the National Ambulatory Care Reporting System (NACRS), Ontario Health Insurance Plan (OHIP) claims, Registered Persons Database (RPDB), Ontario Marginalization Index (ON-Marg), 2021 Census, and CIHI Discharge Abstract Database (DAD), among others. Descriptions of the complete datasets and the variables derived from them are provided in Supplemental Table 2.

### Study Sample

**Figure 1.**
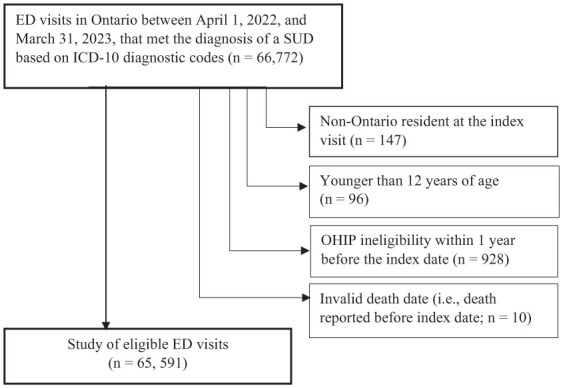
Inclusion criteria of regression analysis involving SUD ED visits.

This study examined data on individuals aged 12 and older who had any SUD-related ED visit (i.e., SUD is not necessarily the primary diagnosis) during the accrual period from April 1, 2022, to March 31, 2023. Restricting to SUD as the main diagnosis would likely exclude many clinically relevant substance-related presentations; therefore, including any-position SUD codes provides a more comprehensive picture of ED utilization among individuals with documented SUD. The ED visit closest to the end of the study period was used as the index visit. The analysis focused on Ontario, Canada’s most populous province, home to 16.1 million people, representing 39% of the country’s total population. In Ontario, in the same timeframe, 3,351,286 unique individuals accessed emergency department services, regardless of presenting condition. Diagnostic codes from the International Classification of Diseases, tenth edition (ICD-10) were used to define SUD ED patients (see Supplemental Table 1), resulting in an initial sample of 66,772 unique patients. Data on diagnostic codes of SUD ED patients were sourced from the NACRS. Exclusions were applied to visits involving non-Ontario residents at the index visit (*n* = 147), individuals younger than 12 years (*n* = 96), those with Ontario Health Insurance Plan (OHIP) ineligibility within 1 year prior to the index date (*n* = 928), and records with invalid death dates (i.e. death reported before the index date; *n* = 10). The final sample of 65,591 unique patients with SUD was included in the study (see Figure 1).

### Sociodemographic Variables

Sociodemographic variables included age, sex, region, and community size. Age was divided into four ranges: 12 to 24, 25 to 44, 45 to 64, and 65 and older. *Sex* was a binary variable coded as either male or female. The six *Ontario Health Regions* (OHR) reflect large administrative areas used for health system planning. The OHRs are grouped into six regions: Central (e.g., Mississauga, Halton Hills, Muskoka Lakes), East (e.g., Pickering, Kingston, Ottawa), North East (e.g., Parry Sound, Sault Ste. Marie, Sudbury), North West (e.g., Thunder Bay, Kenora, Hudson Bay Coast), Toronto (City of Toronto), and West (e.g., Windsor, Waterloo, Niagara).

### Equity-Related Variables

On-Marg (2021 edition; van Ingen & Matheson, 2022) uses the following four dimensions of marginalization coded into quintiles ranging from low marginalization (“Q1”) to high marginalization (“Q5”). All ON-MARG dimensions in this study represent neighborhood-level, not individual-level, characteristics. The *households and dwellings dimension* examines the degree of family and neighborhood stability and cohesiveness (e.g., greater concentration of rented dwellings, higher residential crowding, and more people living alone) from Q1 (‘least marginalized) to Q5 (“most marginalized”). *The material resources dimension* reflects individuals and communities’ challenges in accessing and securing basic necessities such as housing, food, clothing, and education. *The age and labor force* dimension captures the impacts of disability and dependence, reflecting area-level concentrations of individuals without employment income. This includes older adults, children, unpaid workers, and those unable to work due to disability. Lastly, the *racialized and newcomer populations dimension* measures the proportion of non-white, non-Indigenous individuals and newcomers.

### Clinical Variables

*Substance type use at the index visit* categorizes the individual’s SUD class at the ED index visit (e.g., alcohol, opioids, sedatives, stimulants, polysubstance). Each SUD class was a binary variable (1 = yes, 0 = no) derived from the ICD-10 diagnostic codes (Supplemental Table 1). The “Polysubstance” classification follows the ICD-10 F19 code, described as “Mental and behavioural disorders due to multiple drug use and use of other psychoactive substances” (World Health Organization, 1992). Similarly, “Abuse of non-dependence substances” follows the ICD-10 F55 code, which describes the excessive use of psychotropic drugs that do not produce dependence, such as laxatives, antidepressants, and analgesics (WHO, 1992). *Comorbid Mental Illness and Substance-Induced disorders* is a binary variable (1 = yes) and refers to whether an individual experienced at least one hospitalization and/or one or more outpatient physician claims within the past 12 months for a comorbid mental illness and alcohol/drug-induced disorder. *Charlson Comorbidity Index* (CCI) is scored by assigning weighted points to specific comorbid conditions based on severity, with the total score reflecting the overall mortality risk (Charlson et al., 1987). Among the 17 conditions listed on the CCI, Acquired Immunodeficiency Syndrome (AIDS) is an example of a high-severity condition with a score of 6, while dementia scores 1 and is considered a low-severity condition. Based on CCI scores, ICES has coded the index into five categories: No hospitalization (in the last 2 years); 0 (the individual had at least one hospitalization, but their records show that none of the 17 conditions were reported); 1, 2, and CCI ⩾ 3. *Number of Chronic Conditions* captures the total count of documented chronic conditions for each individual, regardless of hospitalization status. Conditions included hypertension, diabetes, mental illness, alcohol/drug-induced disorders, traumatic brain injury, stroke, ischemic heart disease, congestive heart failure, and Parkinsonism.

### Health Service Utilization Variables

*ED frequency* was defined as the number of SUD–related ED visits occurring in the 12 months preceding an individual’s index visit (the most recent SUD-related ED visit). The number of prior visits, excluding the index visit, was categorized as 0 (no prior visits), 1, 2, or 3 or more visits. The outcome was defined as having three or more SUD-related ED visits. This cut-off of three or more visits approximated the 90th percentile of the distribution and was used to define high-frequency ED use. Individuals whose index visit was also their first SUD-related ED visit were classified in the “0 prior visits” group. This categorization was intended to distinguish low- and high-frequency users for analytical purposes. *The number of visits to primary care providers (PCP), the number of visits to psychiatrists, and the number of mental health hospitalizations (e.g., inpatient mental health care, hospitalization for suicidal behavior)* were also collected. *The number of acute care hospitalizations* refers to the total count of times an individual is formally admitted to and discharged from an acute inpatient hospital within a given period. Acute care means necessary treatment for disease or severe illness provided for a short period in a hospital, typically involving active, multidisciplinary management and excluding long-term, chronic, or rehabilitation care. These service utilization and hospitalization variables were coded into four categories: 0 (never hospitalized), 1 (once), 2 (twice), and 3 or more times. *Total ED cost prior to the index date* refers to the patient’s cumulative ED-related costs over the year preceding their index visit. These costs were estimated using the Cost Per Weighted Case methodology (Canadian Institute for Health Information, 2024), which derives individual-level estimates by allocating the total provincial healthcare operational budget, funded by the Ministry of Health and Long-Term Care, across patient visits, based on annual hospital-reported expenditures from all hospitals in Ontario. This costing method follows the Canadian Institute for Health Information’s (CIHI) methodology, which calculates costs based on resource intensity weights (RIWs), case mix groups (CMGs), and standardized annual hospital expense reports to ensure comparability and standardization in Canadian health research (Canadian Institute for Health Information, 2024). This variable was coded into four cost ranges in Canadian dollars (CAD): 0, 1 to 540, 541 to 1,641, and 1,641 and more. These categories were selected based on percentile distributions, with no ED costs at the 25th percentile, 1 to 540 at the 50th percentile, 541 to 1,641 at the 75th percentile, and costs of 1641 CAD or more above the 75th percentile.

### Statistical Analyses

First, descriptive statistics of sociodemographic, equity-related, clinical, and health service utilization variables were conducted and examined across groups based on their frequency of SUD ED visits before the index visit: no visits, one visit, two visits, or three or more visits. Second, logistic regression was conducted to identify factors contributing to three or more ED visits with SUD versus fewer than three visits, with adjusted odds ratios reported as measures of association rather than risk. The outcome variable for the regression model is individuals who visited the ED three or more times for SUD-related issues. Due to the very small sample sizes for tobacco (0.24%), solvent (0.03%), and non-dependent substance abuse (0.05%), each yielding fewer than 10 cases within the 1-, 2-, and ⩾3-visit ED categories, these variables were excluded from the regression analysis. Potential multicollinearity among categorical predictors was assessed using contingency tables and chi-square tests to examine inter-variable associations, and highly correlated variables were removed from the regression model (Wissmann & Toutenburg, 2007). Furthermore, we conducted a complete-case analysis, excluding observations with missing values (5.4%) from the logistic regression models (*n* = 62,018) to minimize potential bias due to missingness. Confidence intervals (CIs, 95%), adjusted odds ratio (aOR), and significance were reported. Regression models were performed using the Statistical Analysis Software (SAS) EG 8.

## Results

Descriptive statistics are reported in Tables 1 to 4, categorized by number of SUD ED visits. Among 65,591 individuals with at least one SUD-related ED visit, 7,885 (12.0%) had three or more SUD-related ED visits in the prior 12 months, compared to 43,211 (65.9%) with no prior SUD-related ED visits, 10,297 (15.7%) with one, and 4,198 (6.4%) with two prior visits. Regarding *sociodemographic factors*, the distribution of visits varies across demographic categories (Table 1). For instance, younger individuals aged 12 to 44 accounted for 64.6% of total visits, with the 25 to 44 age group comprising the largest proportion at 44.9%. Across all *equity-related factors* (Table 2), individuals in more marginalized categories (e.g. unstable and disadvantaged residential and family structures, low access to material resources, high dependency, and high diversity areas) tend to have higher frequencies of SUD ED visits. *Clinical factors* reveal significant contrasts, particularly regarding chronic conditions and mental health (Table 3). About 97.6% of individuals with 3+ prior ED visits had one or more chronic conditions, compared to 73.5% of those with no prior ED visits. Substance use patterns varied, with alcohol being the most prevalent substance (51.42% of visits), followed by sedatives (11.7%) and opioids (8.4%). Healthcare utilization data showed stark contrasts (Table 4). For instance, among those with no prior ED visits, 5.85% had a mental health hospitalization, compared to 36.95% of those with two visits and 53.21% of those with 3+ visits. Acute care hospitalizations followed a similar pattern: 14.08% of individuals with no prior ED visit had a prior acute hospitalization, compared with 68.15% of individuals with 3+ ED visits. Standardized differences for group comparisons (0 visits vs. 1, 2, and 3+ visits) along with overall *p*-values are reported in Supplemental Table 3.

**Table 1.** Sociodemographic Characteristics of SUD ED Visitors Based on the Number of ED Visits.

Characteristic	Value	Total, *n*	%	0 visits, *n*	%	1 visit, *n*	%	2 visits, *n*	%	3+ visits, *n*	%	χ^2^ (*p*-value)
Age groups (years) at index	12–24	12,911	19.68	9,848	22.79	1,685	16.36	599	13.43	779	10.22	<.001
	25–44	29,450	44.90	17,745	41.07	5,065	49.19	2,367	53.07	4,273	56.05	
	45–64	16,978	25.88	10,917	25.26	2,735	26.56	1,177	26.39	2,149	28.19	
	65 or older	6,252	9.53	4,701	10.88	812	7.89	317	7.11	422	5.54	
Sex on RPDB*	Female	23,804	36.29	16,164	37.41	3,533	34.31	1,537	34.46	2,570	33.71	<.001
	Male	41,787	63.71	27,047	62.59	6,764	65.69	2,923	65.54	5,053	66.29	
Ontario Health Region^a^ at index	Central	11,216	17.10	7,825	18.11	1,580	15.34	683	15.31	1,128	14.80	<.001
	East	12,233	18.65	8,504	19.68	1,882	18.28	746	16.73	1,101	14.44	
	North East	5,070	7.73	3,163	7.32	847	8.23	376	8.43	684	8.97	
	North West	4,563	6.96	2,358	5.46	879	8.54	388	8.70	938	12.30	
	Toronto	12,892	19.66	8,022	18.56	1,990	19.33	1,003	22.49	1,877	24.62	
	West	19,617	29.91	13,339	30.87	3,119	30.29	1,264	28.34	1,895	24.86	

* RPDB = Registered Persons Database (biological sex).

a Ontario results are grouped by the six Ontario Health regions: Central (e.g., Mississauga, Halton Hills, Muskoka Lakes); East (e.g., Pickering, Kingston, Ottawa); North East (e.g., Parry Sound, Sault Ste. Marie, Sudbury); North West (e.g., Thunder Bay, Kenora, Hudson Bay Coast; Toronto (City of Toronto); West (e.g., Windsor, Waterloo, Niagara).

**Table 2.** Equity-related characteristics of SUD ED visitors based on the number of ED visits. (ONMARG* 2021 edition).

Characteristic	Value	Total, *n*	%	0 visits, *n*	%	1 visit, *n*	%	2 visits, *n*	%	3+ visits, *n*	%	χ^2^ (*p*-value)
Households and dwellings	Q1, Least disadvantaged	7,215	11.00	5,320	12.31	968	9.40	387	8.68	540	7.08	<.001
	Q2, Low	8,143	12.41	5,829	13.49	1,174	1.40	456	10.22	684	8.97	
	Q3, Moderate	9,884	15.07	6,835	15.82	1,483	14.40	631	14.15	935	12.27	
	Q4, High	13,996	21.34	9,318	21.56	2,191	21.28	942	21.12	1,545	20.27	
	Q5, Most disadvantaged	22,984	35.04	14,097	32.62	3,872	37.60	1,747	39.17	3,268	42.87	
	Missing data	3,369	5.14	1,812	4.19	609	5.91	297	6.66	651	8.54	
Material resources	Q1, Least deprived	8,714	13.29	6,129	14.18	1,213	11.78	520	11.66	852	11.18	<.001
	Q2, Low	9,958	15.18	6,980	16.15	1,470	14.28	588	13.18	920	12.07	
	Q3, Moderate	10,610	16.18	7,249	16.78	1,617	15.70	680	15.25	1,064	13.96	
	Q4, High	12,486	19.04	8,261	19.12	1,986	19.29	855	19.17	1,384	18.16	
	Q5, Most deprived	20,454	31.18	12,780	29.58	3,402	33.04	1,520	34.08	2,752	36.10	
	Missing data	3,369	5.14	1,812	4.19	609	5.91	297	6.66	651	8.54	
Age and labor force	Q1, Least dependent	15,093	23.01	10,097	23.37	2,287	22.21	962	21.57	1,747	22.92	<.001
	Q2, Low	12,483	19.03	8,262	19.12	1,959	19.02	869	19.48	1,393	18.27	
	Q3, Moderate	11,213	17.10	7,511	17.38	1,761	17.10	721	16.17	1,220	16.00	
	Q4, High	10,727	16.35	7,135	16.51	1,688	16.39	710	15.92	1,194	15.66	
	Q5, Most dependent	12,706	19.37	8,394	19.43	1,993	19.36	901	20.20	1,418	18.60	
	Missing data	3,369	5.14	1,812	4.19	609	5.91	297	6.66	651	8.54	
Racialized and newcomer populations	Q1, Least diverse	12,180	18.57	8,102	18.75	1,950	18.94	802	17.98	1,326	17.39	<.001
	Q2, Low	11,588	17.67	7,865	18.20	1,772	17.21	742	16.64	1,209	15.86	
	Q3, Moderate	12,310	18.77	8,086	18.71	1,983	19.26	826	18.52	1,415	18.56	
	Q4, High	12,888	19.65	8,485	19.64	1,975	19.18	907	20.34	1,521	19.95	
	Q5, Most diverse	13,256	20.21	8,861	20.51	2,008	19.50	886	19.87	1,501	19.69	
	Missing data	3,369	5.14	1,812	0.19	609	5.91	297	6.66	651	8.54	

* ON-MARG quintiles represent neighborhood-level marginalization, derived from census data and linked using residential postal codes; they do not capture individual-level socioeconomic position or racialized identity.

**Table 3.** Clinical Characteristics of SUD ED Visitors Based on the Number of ED Visits.

Characteristic	Value	Total, *n*	%	0 visits, *n*	%	1 visit, *n*	%	2 visits, *n*	%	3+ visits, *n*	%	χ^2^ (*p*-value)
Comorbidity last 12 months^a^	No	20,169	30.75	17,461	40.41	1,836	17.83	467	0.47	405	5.31	<.001
	Yes	45,422	69.25	25,750	59.59	8,461	82.17	3,993	89.53	7,218	94.69	
Substance Type use at the index Visit at index	Alcohol	37,147	56.63	24,930	57.69	5,423	52.67	2,384	53.45	4,410	57.85	<.001
	Cannabis	7,406	11.29	5,843	13.52	915	8.89	306	6.86	342	4.49	
	Cocaine	3,170	4.83	2,119	4.90	559	5.43	214	4.80	278	3.65	
	Hallucinogen	294	0.45	244	0.56	26	0.25	14	0.31	10	0.13	
	Opioid	6,641	10.12	3,678	8.51	1,404	13.64	597	13.39	962	12.62	
	Polysubstance^b^	7,237	11.03	4,353	10.07	1,312	12.74	604	13.54	968	12.70	
	Sedative	511	0.78	362	0.84	79	0.77	32	0.72	38	0.50	
	Solvent	21	0.03	11	0.03	1–5*	1–5*	1–5*	1–5*	1–5*	1–5*	
	Stimulant	2,969	4.53	1,495	3.46	568	5.52	299	6.70	607	7.96	
	Tobacco	160	0.24	146	0.34	6–10*	6–10*	5–9*	5–9*	1–5*	1–5*	
	ND substance^c^	35	0.05	30–34*	30–34*	1–5*	1–5*	1–5*	1–5*	3–7*	3–7*	
Charlson Comorbidity Index, lifetime	0	45,644	69.59	30,595	70.80	7,216	70.08	3,040	68.16	4,793	62.88	<.001
	1	6,655	10.15	3,613	8.36	1,142	11.09	576	12.91	1,324	17.37	
	2	2,457	3.75	1,409	3.26	401	3.89	222	4.98	425	5.58	
	3+	3,492	5.32	1,832	4.24	627	6.09	314	7.04	719	9.43	
	0 Hospitalization	7,343	11.20	5,762	13.33	911	8.85	308	6.91	362	4.75	
Number of chronic conditions	None	12,967	19.77	11,470	26.54	1,062	10.31	255	5.72	180	2.36	<.001
	One	32,893	50.15	20,795	48.12	5,773	56.06	2,482	55.65	3,843	50.41	
	Two	14,220	21.68	8,040	18.61	2,527	24.54	1,220	27.35	2,433	31.92	
	Three or more	5,511	8.40	2,906	6.73	935	9.08	503	11.28	1,167	15.31	

a Comorbid mental illness and substance-induced disorders in the last 12 months.

b Multi-substance or other psychoactive substances.

c Abuse of non-dependence substances.

* In accordance with ICES’ privacy policy, small cell counts (<6) are suppressed to reduce the risk of participant identification.

**Table 4.** Healthcare Utilization Characteristics of SUD ED Visitors Based on the Number of ED Visits.

Characteristic	Value	Total, *n*	%	0 visits, *n*	%	1 visit, *n*	%	2 visits, *n*	%	3+ visits, *n*	%	χ^2^ (*p*-value)
Total ED cost prior to the index date (in CAD)	$ 0	20,543	31.32	20,543	47.54	0	0.00	0	0.00	0	0.00	<.001
	$ 1–540	12,288	18.73	9,887–9,891^a^		2,321	22.54	75	1.68	1–5^a^		
	$ 541–1,621	16,365	24.95	9,001–9,005^a^		4,920	47.78	1,883	42.22	557–561^a^		
	More than $ 1,621	16,395	25.00	3,776	8.74	3,056	29.68	2,502	56.10	7,061	92.63	
Number of visits to the Primary care practitioner	None	15,709	23.95	11,684	27.04	2,190	21.27	769	17.24	1,066	13.98	<.001
	One	8,189	12.48	5,646	13.07	1,254	12.18	493	11.05	796	10.44	
	Two	6,545	9.98	4,540	10.51	970	9.42	407	9.13	628	8.24	
	Three or more	35,148	53.59	21,341	49.39	5,883	57.13	2,791	62.58	5,133	67.34	
Number of visits to a psychiatrist	None	50,706	77.31	36,435	84.32	7,208	70.00	2,815	63.12	4,248	55.73	<.001
	One	4,297	6.55	2,045	4.73	908	8.82	504	11.30	840	11.02	
	Two	2,486	3.79	1,167	2.70	542	5.26	281	6.30	496	6.51	
	Three or more	8,102	12.35	3,564	8.25	1,639	15.92	860	19.28	2,039	26.75	
Number of mental health hospitalization within 12 months of the index visit	None	54,888	83.68	40,685	94.15	7,824	75.98	2,812	63.05	3,567	46.79	<.001
	One	6,539	9.97	1,883	4.36	1,850	17.97	1,048	23.50	1,758	23.06	
	Two	2,187	3.33	402	0.93	397	3.86	404	9.06	984	12.91	
	Three or more	1,977	3.01	241	0.56	226	2.19	196	4.39	1,314	17.24	
Number of acute care hospitalizations within 12 months of the index visit	None	47,803	72.88	37,125	85.92	6,169	59.91	2,081	46.66	2,428	31.85	<.001
	One	10,087	15.38	4,304	9.96	2,737	26.58	1,255	28.14	1,791	23.49	
	Two	3,718	5.67	1,062	2.46	797	7.74	662	14.84	1,197	15.70	
	Three or more	3,983	6.07	720	1.67	594	5.77	462	10.36	2,207	28.95	

a In accordance with ICES’ privacy policy, small cell counts (<6) are suppressed to reduce the risk of patient identification.

Table 5 shows factors associated with three or more SUD ED visits. Young adults aged 25 to 44 consistently emerge as a strong factor associated with frequent ED visits for SUD (age 25-–44 vs. 65+ aOR: 2.56, 95% CI: [2.22, 2.94], *p* < .01), highlighting the vulnerability of this age group. Men are also more likely to have three or more ED visits (aOR: 1.38, 95% CI: [1.29, 1.48], *p* < .001). Regionally, the North West consistently shows the strongest association with ED visits (aOR: 1.67, 95% CI: [1.42, 1.97], *p* < .001), while the Central and West regions show decreased likelihood compared to Toronto. Area-level equity-related indices, such as unstable housing (Q5: aOR: 1.30, 95% CI: [1.13, 1.49], *p* < .001), are associated with a higher likelihood of frequent ED visits. The racialized and newcomer population was not significantly associated with frequent SUD ED visits. Individuals with comorbid mental illnesses are among the frequent users of ED (aOR: 2.30, 95% CI: [1.00, 2.64], *p* < .001), reinforcing the interplay between mental health and substance use. The regression model demonstrated good overall performance, with a c-statistic (AUROC) indicating strong discrimination and a Hosmer–Lemeshow test supporting adequate calibration. Model fit indices were: AIC = 22,524.6, −2 Log Likelihood = 22,418.6, *R*-square = .289, and max-rescaled *R*-square = .573. These results indicate the model offers robust discriminatory power and stable estimation of adjusted odds ratios.

**Table 5.** Adjusted Associations (OR, 95% CI) Between Sociodemographic, Marginalization, Clinical and Health-Service Utilization Variables and Three or More ED Visits Versus Fewer than Three Visits (*n* = 62,052).

Variable	Total, *n*	%	OR	95% C.I	*p*
Sociodemographic					
*Sex*					
Female	23,804	36.29	Ref		
Male	41,787	63.71	1.38	1.29–1.48	<.001
*Age groups*					
65 and over	6,252	9.53	Ref		
12–24	12,911	19.68	1.37	1.16–1.63	<.001
25–44	29,450	44.90	2.56	2.22–2.94	<.001
45–64	16,978	25.88	1.98	1.73–2.28	<.001
*Ontario health region*					
Toronto	12,892	19.66	Ref		
Central	11,216	17.10	0.82	0.73–0.91	<.001
East	12,233	18.65	0.66	0.59–0.74	<.001
North East	5,070	7.73	0.90	0.77–1.05	
North West	4,563	6.96	1.67	1.42–1.97	<.001
West	19,617	29.91	0.68	0.61–0.75	<.001
Marginalization					
*Households and dwellings*					
Q1, least unstable	7,215	11.00	Ref		
Q2	8,143	12.41	1.04	0.89–1.21	
Q3	9,884	15.07	1.15	0.99–1.33	
Q4	13,996	21.34	1.21	1.05–1.40	<.01
Q5, most unstable	22,984	35.04	1.30	1.13–1.49	<.001
*Material resources*					
Q1, least deprived	8,714	13.29	Ref		
Q2	9,958	15.18	0.97	0.85–1.11	
Q3	10,610	16.18	0.98	0.86–1.12	
Q4	12,486	19.04	0.96	0.85–1.09	
Q5, most deprived	20,454	31.18	0.99	0.88–1.12	
*Age and labor force*					
Q1, least dependent	15,093	23.01	Ref		
Q2	12,483	19.03	0.87	0.78–0.96	<.01
Q3	11,213	17.10	0.87	0.78–0.96	<.01
Q4	10,727	16.35	0.90	0.81–1.00	
Q5, most dependent	12,706	19.37	0.81	0.72–0.90	<.001
*Racialized and newcomer population*					
Q1, highest ethnic diversity	15,093	23.01	Ref		
Q2	11,588	17.67	1.04	0.93–1.16	
Q3	12,310	18.77	1.02	0.91–1.15	
Q4	12,888	19.65	1.12	0.99–1.26	
Q5, lowest ethnic diversity	13,256	20.21	1.01	0.88–1.15	
Clinical					
*Charlson index category*					
3+	7,343	11.20	Ref		
No hospitalization	45,644	69.59	1.03	0.85–1.25	
0	6,655	10.15	1.03	0.91–1.18	
1	2,457	3.75	1.27	1.10– 1.46	<.001
2	3,492	5.32	0.96	0.81–1.14	
*Comorbid mental illness and substance-induced disorders*					
No	20,169	30.75	Ref		
Yes	45,422	69.25	2.30	2.00–2.64	
*Substance type use at index visit*					
Alcohol	37,147	56.82	Ref		
Cannabis	7,406	11.33	0.23	0.20–0.26	<.001
Cocaine	3,170	4.85	0.41	0.35–0.49	<.001
Hallucinogen	294	0.45	0.22	0.10–0.46	<.001
Opioid	6,641	10.16	0.55	0.50–0.61	<.001
Polysubstance^a^	7,237	11.07	0.47	0.43–0.52	<.001
Sedative	511	0.78	0.27	0.18–0.39	<.001
Stimulant	2,969	4.57	0.65	0.57–0.74	<.001
Health-service utilization					
*# Of visits to PCP*					
None	15,709	23.95	Ref		
Once	8,189	12.48	1.01	0.88–1.15	
Twice	6,545	9.98	0.84	0.72–0.97	<.05
Three or more times	35,148	53.59	0.98	0.88–1.08	
*# Of mental health hospitalizations*					
None	54,888	83.68	Ref		
One	6,539	9.97	2.28	2.04–2.53	<.001
Two	2,187	3.33	3.38	2.91–3.92	<.001
Three or more	1,977	3.01	4.36	3.68–5.17	<.001
*# Of visits to psychiatrists*					
None	50,706	77.31	Ref		
Once	4,297	6.55	1.06	0.95–1.19	
Twice	2,486	3.79	1.04	0.90–1.19	
Three or more times	8,102	12.35	1.10	1.01–1.21	<.05
*# Of acute care hospitalizations*					
None	47,803	72.88	Ref		
Once	10,087	15.38	0.40	0.35–0.46	<.001
Twice	3,718	5.67	0.69	0.60–0.80	<.001
Three or more times	3,983	6.07	0.69	0.60–0.80	<.001

a Multi-Substance or Other Psychoactive Substances.

## Discussion

This study offers a comprehensive analysis of patients with SUD-related ED visits in Ontario (2022–2023), examining sociodemographic characteristics, equity-related determinants, clinical profile, and patterns of health service utilization. By incorporating broader measures, including equity-related factors, this research provides a more comprehensive understanding of the factors associated with frequent ED utilization. Our findings underscore the multifaceted challenges faced by high-frequency ED patients with SUD, who often embody a complex intersection of clinical needs, socioeconomic vulnerabilities, and systemic shortcomings. These insights contribute valuable evidence to inform policy and practice to improve care delivery for individuals with SUD.

Consistent with the existing literature, current findings confirm that younger adults, particularly those aged 25 to 44 years and male sex, are associated with SUD-related ED visits (Fleury et al., 2019; Kim, Weekes et al., 2023; Lavergne et al., 2022; Moe et al., 2022). ED visit rates are particularly high in Northwestern Ontario, where geographic remoteness, limited healthcare infrastructure, and socioeconomic challenges contribute to poorer outcomes (Ontario Health, 2025). While Central and Toronto are urban cores with concentrated specialized care, the northern regions are vast and sparsely populated, with many communities located far from hospitals (Ontario Ministry of Health, 2025). In another study in Ontario, rural areas had a higher risk of post-discharge mortality in patients with alcohol use disorder, mediated by reduced access to mental health and addiction services (Friesen et al., 2023). Strategies such as telehealth services and improved transportation options can help address access issues in remote areas.

Aligned with prior studies, households and dwellings marginalization, including frequent moves, crowded or non-family living arrangements, and lower rates of home ownership, was associated with frequent SUD-related ED visits (Bell et al., 2024; Kim, Weekes et al., 2023; van Ingen & Matheson, 2022). Unstable housing can disrupt access to preventative and primary health care, thereby affecting SUD outcomes such as prognosis and mortality (Bell et al., 2024; Lin et al., 2024; Morton, 2019; Sarkar et al., 2021). These effects likely emerge through interactions among individual psychological and behavioral factors (e.g., coping styles, help-seeking behaviors), environmental and social contexts (e.g., social support networks), and system-level influences (e.g., access to patient-centered addiction services; Bell et al., 2024; Fox et al., 2019; Lin et al., 2024; Morton, 2019; Sarkar et al., 2021). This may indicate the importance of addressing individual, social, and healthcare system factors to improve outcomes for individuals affected by SUD.

Residing in an ethnically dense neighborhood was not significantly associated with frequent SUD-related ED utilization. However, as this analysis used neighborhood-level data, the results should be interpreted cautiously, as they do not reflect individual-level patterns among immigrant or racialized populations. Consistent with prior research, the “healthy immigrant effect” suggests that immigrants, including refugees, tend to have lower rates of SUD compared to non-immigrants (Salas-Wright et al., 2018; Salas-Wright & Vaughn, 2014). Likewise, large-scale studies show that ethnoracial groups generally have lower overall rates of SUD, contradicting stereotypes that link race or ethnicity with substance misuse (Alvarez et al., 2019; Barr et al., 2022). Despite lower prevalence, racialized individuals with SUD often face disproportionately worse outcomes and barriers to culturally safe care (Farahmand et al., 2020; Jackson et al., 2022). For instance, SUD-related ED visits are predominantly among non-Hispanic White patients (approximately 60%), while Asian/Pacific Islander and Hispanic individuals account for substantially smaller proportions (1.1% and 12.3%, respectively; Owens & Moore, 2023). Black patients represent about 20.6% of visits but are 53% less likely than White patients to receive an immediate or emergent triage level, underscoring systemic inequities (Goldfarb et al., 2025). These findings challenge racialized explanations of SUD patterns and instead emphasize structural and contextual determinants of care. The absence of a significant association with the racialized and newcomer marginalization index does not imply an absence of need; rather, it reinforces the imperative for equitable access and culturally responsive interventions. Efforts must focus on dismantling systemic barriers and developing tailored, equity-oriented approaches that address social, linguistic, and cultural determinants of health (Banks et al., 2023; Farahmand et al., 2020; Fox et al., 2019; Jackson et al., 2022).

The significant contribution of comorbid mental illnesses, chronic diseases, and substance-induced disorders to increased ED visits is consistent with prior studies (Curran et al., 2003, 2008; Gentil et al., 2021; Wu et al., 2018). These co-occurring conditions often exacerbate the severity and complexity of SUDs, increasing the likelihood of acute care needs. Integrated care models that address the interplay between co-occurring conditions may be needed. Further, alcohol was most strongly associated with higher ED visit frequencies (Covino et al., 2021; Kim, Rajack et al., 2023; Kim, Weekes et al., 2023; Moe et al., 2022). Similarly, Kepner et al. (2024) found that individuals with substance-related diagnoses, particularly alcohol, opioid, and stimulant use disorders, face substantially increased odds of ED utilization, with risks amplified by social disadvantage and comorbid psychiatric conditions. This finding highlights the pervasive impact of alcohol-related disorders, which are known to frequently result in acute episodes requiring emergency care, such as alcohol intoxication, withdrawal symptoms, or injuries associated with intoxication. Also, it is worth noting that poly-substance use significantly contributed to frequent ED visits, with individuals presenting multiple substance types at their index visit showing markedly higher utilization (Covino et al., 2021).

Individuals with a history of frequent hospitalizations, whether for mental health or acute care, were strongly associated with frequent SUD ED visits (Krieg et al., 2016). This finding highlights that frequent ED users often represent a complex population characterized by overlapping clinical vulnerabilities (Van Den Heede & Van de Voorde, 2016) and the importance of the continuity of care (van Walraven et al., 2010). Additionally, high prior ED costs and frequent psychiatric consultations emphasize the substantial resource burden required to manage individuals with recurrent substance use–related emergencies (Beckerleg & Hudgins, 2022). This pattern indicates repeated reliance on costly emergency care, driven by patients’ complex needs, including comorbid mental health conditions and limited access to preventive services. High ED users typically need extensive diagnostics, specialist care, and crisis interventions, resulting in greater health expenditures. Evidence also links high ED use to factors such as homelessness, injection drug use, and comorbidities, emphasizing the need for integrated interventions that address both medical and psychosocial complexity (Armoon et al., 2023; Gramaglia et al., 2025). Integrated care approaches, such as Collaborative Care Models (CCM) and co-located services, combine mental health, primary care, and addiction treatment for comprehensive management (Ee et al., 2020; Lake & Turner, 2017; Reist et al., 2022). CCM involves coordinated care by primary and behavioral health providers, addressing the full spectrum of patient needs. These models can lower healthcare costs and improve outcomes by tackling both immediate and underlying drivers of ED visits(Ee et al., 2020; Lake & Turner, 2017; Lee et al., 2010; Morley et al., 2018; Turner et al., 2015; Van Den Heede & Van de Voorde, 2016).

While these findings provide valuable insights, they are based on cross-sectional data, limiting the ability to draw causal inferences. The healthcare cost analyses were also confined to ED expenses, excluding other healthcare system components, such as primary care and outpatient services. This may result in underestimating the total economic burden of frequent ED utilization. Marginalization factors were assessed at the area level rather than the individual level, reducing the precision of insights into how these factors directly influence ED use. Furthermore, the data only indicate SUD-related ED visits without details on the specific medical issues for which treatment was sought. Also, the variable “sex” is based on biological sex, with no information on gender identity available in these data sources. This limitation may mask underlying gender-related disparities in substance use disorder care pathways. Finally, the cross-sectional design relies on retrospective administrative data, which may be subject to coding inaccuracies, and the 1-year look-back period may not fully capture longer-term utilization patterns. The reported odds ratios may overestimate the strength of association for this relatively common outcome and should not be interpreted as risk or prevalence ratios.

Despite these limitations, this study uniquely integrates multiple population-level data sources to indicate how social marginalization, health complexity, and service use intersect among individuals with SUD-related ED visits. Frequent ED use was most strongly associated with young adulthood, male sex, households and dwellings marginalization, comorbid mental illness, alcohol use, and prior high healthcare utilization, suggesting that socioeconomic and clinical vulnerabilities outweigh ethnicity or newcomer status as predictors. Addressing these challenges requires expanding access to preventive and rapid addiction services, such as RAAM clinics, which Ontario studies show reduce emergency department visits among people with substance use disorders (Corace et al., 2023; Wiercigroch et al., 2020), implementing integrated care for polysubstance use and chronic conditions, strengthening referral and continuity systems, and ensuring equitable telehealth and community-based supports across urban and rural areas.

## Supplemental Material

sj-docx-1-isp-10.1177_00207640261419135 – Supplemental material for Equity-Related Determinants of Frequent Emergency Department Visits for Substance Use Disorders: A Population-Based Study in Ontario, Canada Supplemental material, sj-docx-1-isp-10.1177_00207640261419135 for Equity-Related Determinants of Frequent Emergency Department Visits for Substance Use Disorders: A Population-Based Study in Ontario, Canada by Soyeon Kim, Kenneth P. Fung, Natalie Rajack, Florence Tang, Aditi Patrikar, Kinwah Fung, Erik L. Friesen, Paul Kurdyak and Bernard Le Foll in International Journal of Social Psychiatry
