# Supplementary material for: Equity-Related Determinants of Frequent Emergency Department Visits for Substance Use Disorders: A Population-Based Study in Ontario, Canada
Source: Int J Soc Psychiatry. 2026 Feb 28;72(5):1426–38. doi: 10.1177/00207640261419135 (PMC13478429; doi:10.1177/00207640261419135)
Supplement: Supplementary file 1 [file sj-docx-1-isp-10.1177_00207640261419135.docx]

## Supplementary Table 1: ICD-10 diagnostic codes for substance use and related disorders

| **Substance/Condition type** | **ICD-10 Code** |
| --- | --- |
| Alcohol use disorder | F10 |
| Opioid use disorder | F11 |
| Cannabis Use Disorder | F12 |
| Sedative/Hypnotic Use Disorder | F13 |
| Cocaine Use Disorder | F14 |
| Stimulant Use Disorder | F15 |
| Hallucinogen Use Disorder | F16 |
| Tobacco Use Disorder | F17 |
| Solvent Use Disorder  Multi-Substance or Other Psychoactive Substances  Abuse of Non-dependence Substances | F18  F19  F55 |

## Supplementary Table 2: Data Source and Variables

| **Data Source (abbr.)** | **Description** | **Variables** |
| --- | --- | --- |
| National Ambulatory Care Reporting System (NACRS) | Collects service utilization data for hospital- and community-based ambulatory care such as emergency departments (ED), day surgery and other ambulatory services | - ED frequency - Total ED healthcare costs prior to ED index date - Substance type use at index visit - Number of types of substances - Number of chronic conditions - Discharge disposition - Triage level - Referral source |
| Ontario Health Insurance Program (OHIP) data | Contains physician-level and patient-level data including (but not limited to) sociodemographic, service utilization, and payment information of physicians and service utilization information of physicians’ patients (categorized by sex and age group) | - Number of visits to PCP - Number of visits to psychiatrists - Number of chronic conditions |
| Registered Persons Database (RPDB) | Collects basic demographic information on persons registered under OHIP | - Age groups - Sex in RPDB |
| Institutional Information System and Master Numbering System (INST. MNS) dataset | This linked dataset contains data about Ontario health care institutions funded by the Ministry of Health and Long-Term Care from the Institution Information System. In addition, this dataset links data from the Master Numbering System, which contains information about each institution's number, name, location, type, and the time range it was in the MNS. | - Ontario Health Region |
| The Local Health Integrated Network (LHIN) database | Collects data on regional health authorities in Ontario, responsible for overseeing the administration of public healthcare services | - Ontario Health Region |
| Same day surgery (SDS) database | Contains data on same-day surgical procedures | - Charlson Index Category |
| Ontario Marginalization Index (ONMARG) | It contains Ontario data on a wide range of demographic indicators that are categorized into four dimensions of marginalization: households and dwellings, material resources, age and labor force, and racialized and newcomer populations. | - Household and Dwellings Quintile - Material Resources Quintile - Age and Labour Force Quintile - Racialized and Newcomer population Quintile |
| The Canadian Institute for Health Information’s Discharge Abstract Database (CIHI-DAD) | Collects data on day surgery encounters and hospital discharges (including transfers, sign-outs, and deaths) from acute health facilities in all provinces except Quebec | - Number of acute care hospitalizations (one-year lookback) - Charlson Index Category - Mental illness and alcohol/drug-induced disorders (2-year lookback) |
| Ontario Mental Health Reporting System (OMHRS) | Collects data on individuals admitted to mental health hospitals in Ontario | - Number of mental health hospitalizations (12 months prior to index) - Number of chronic conditions |

**Supplementary Table 3.** Standardized differences between the groups and p-values

| Characteristic | Value | 0 visit vs.  1 visit | 0 visit vs.  2 visits | 0 visit vs.  3+ visits |
| --- | --- | --- | --- | --- |
| Age groups (years) | 12-24  25-44  45-64  65 or older | 0.162  0.164  0.030  0.103 | 0.245  0.242  0.026  0.132 | 0.344  0.303  0.066  0.196 |
| Sex on RPDB* | Female  Male | 0.065  0.065 | 0.061  0.061 | 0.077  0.077 |
| Ontario Health Region | Central  East  North East  North West  Toronto  West | 0.074  0.036  0.034  0.121  0.019  0.013 | 0.075  0.077  0.041  0.127  0.097  0.055 | 0.089  0.140  0.060  0.242  0.148  0.134 |
| Households and Dwellings | Q1, Crowded dwelling  Q2, Below average  Q3, Average space  Q4, Above average  Q5, Very spacious  Missing Data | 0.094  0.063  0.040  0.007  0.104  0.079 | 0.119  0.101  0.047  0.011  0.137  0.109 | 0.177  0.143  0.102  0.032  0.213  0.179 |
| Material Resources | Q1, Low access  Q2, Below average  Q3, Average access  Q4, Above average  Q5, High access  Missing data | 0.072  0.052  0.029  0.004  0.075  0.079 | 0.075  0.084  0.042  0.001  0.097  0.109 | 0.090  0.118  0.078  0.025  0.139  0.179 |
| Age and Labor Force | Q1, High dependency  Q2, Above average  Q3, Average dependency  Q4, Below average  Q5, Low dependency  Missing data | 0.028  0.002  0.007  0.003  0.002  0.079 | 0.043  0.009  0.033  0.016  0.019  0.109 | 0.011  0.022  0.037  0.023  0.021  0.179 |
| Racialized and Newcomer Populations | Q1, High diversity  Q2, Above average  Q3, Average diversity  Q4, Below average  Q5, Low diversity  Missing data | 0.005  0.026  0.014  0.012  0.025  0.079 | 0.020  0.041  0.005  0.018  0.016  0.109 | 0.035  0.062  0.004  0.008  0.020  0.179 |
| Comorbid Mental Illness and Substance-Induced disorders | No  Yes | 0.513  0.513 | 0.732  0.732 | 0.920  0.920 |
| Substance Type use at the index visit | Alcohol  Cannabis  Cocaine  Hallucinogen  Opioid  Polysubstance^a^  Sedative  Solvent  Stimulant  Tobacco  ND substances^b^ | 0.101  0.147  0.024  0.049  0.164  0.084  0.008  0.007  0.099  0.063  0.03 | 0.085  0.222  0.005  0.038  0.157  0.108  0.014  0.019  0.148  0.042  0.022 | 0.003  0.32  0.062  0.074  0.134  0.083  0.042  0.008  0.195  0.073  0.013 |
| Charlson Comorbidity Index | 0  1  2  3+  No Hospitalization | 0.016  0.092  0.034  0.084  0.143 | 0.057  0.148  0.086  0.122  0.214 | 0.169  0.271  0.113  0.207  0.303 |
| Number of chronic conditions | None  One  Two  Three or more | 0.428  0.159  0.145  0.087 | 0.590  0.151  0.209  0.160 | 0.732  0.046  0.310  0.277 |
| Total ED cost prior to the index date (in CAD) | $ 0  $ 1-540  $ 541-1621  More than $ 1621 | 1.346  0.008  0.592  0.551 | 1.346  0.683  0.473  1.173 | 1.346  0.769  0.395  3.083 |
| Number of visits to the Primary care practitioner | None  One  Two  Three or more | 0.135  0.027  0.036  0.156 | 0.238  0.062  0.046  0.268 | 0.328  0.082  0.078  0.370 |
| Number of visits to the Psychiatrist | None  One  Two  Three or more | 0.346  0.163  0.131  0.237 | 0.496  0.244  0.174  0.324 | 0.657  0.235  0.182  0.502 |
| Number of mental health hospitalization within 12 months of the index visit | None  One  Two  Three or more | 0.527  0.443  0.192  0.141 | 0.820  0.575  0.380  0.249 | 1.215  0.565  0.486  0.613 |
| Number of acute care hospitalizations within 12 months of the index visit | None  One  Two  Three or more | 0.612  0.440  0.242  0.218 | 0.913  0.476  0.452  0.372 | 1.315  0.369  0.474  0.819 |

*Statistically significant differences were observed across all variables, with overall p-values < .001.

**^a^** = Multi-Substance or Other Psychoactive Substances; ^b^ = Abuse of non-dependence substances *RPDB=Registered Persons Database (biological sex)
